# Assessing skin disease and associated health-related quality of life in a rural Lao community

**DOI:** 10.1186/s12895-018-0079-8

**Published:** 2018-12-04

**Authors:** C. I. Wootton, S. Bell, A. Philavanh, K. Phommachack, M. Soukavong, S. Kidoikhammouan, S. L. Walker, M. Mayxay

**Affiliations:** 1 Lao-Oxford-Mahosot Hospital-Wellcome Trust Research Unit (LOMWRU), Microbiology laboratory, Mahosot Hospital, Vientiane, Laos; 2 Dermatology Department, Lincoln County Hospital, Lincoln, UK; 3 National Dermatology Centre, Ministry of Health, Vientiane, Laos; 4 Department of Hygiene and Health Promotion, Ministry of Health, Vientiane, Laos; 5 Faculty of Basic Sciences, University of Health Sciences, Vientiane, Laos; 6 London School of Hygiene and Tropical Medicine, London, UK; 7 Faculty of Postgraduate Studies, University of Health Sciences, Ministry of Health, Vientiane, Laos; 8 Centre for Tropical Medicine and Global Health, Nuffield Department of Medicine, Churchill Hospital, Oxford, UK

**Keywords:** Prevalence, Health related quality of life, Scabies, Tinea, DLQI, Cercarial dermatitis, Eczema

## Abstract

**Background:**

Skin diseases are common and often have an impact on an individual’s health-related quality of life. In rural communities where access to healthcare may be limited and individuals rely on farming for food and income, the impact of skin diseases may be greater.

The objectives for this study were to perform an assessment of skin disease prevalence in a rural village in Laos and assess the associated impact of any skin disease found using the Dermatology Life Quality Index (DLQI).

**Methods:**

A rural village was purposively selected and 340 participants examined by dermatologists over a four day period. Brief questionnaires were performed, followed by full body skin examinations and DLQI questionnaires completed were relevant. The data were analysed using chi square and Wilcoxon signed rank tests.

**Results:**

One hundred and eighty-one participants were found to have a skin disease (53%). The six most common skin diseases were: eczema (22%), dermatophyte infections (19%), acne (10%), scabies infestation (9%), melasma (8%) and pityriasis versicolor (4%). Just over half of those with skin disease (51%) completed the DLQI, with scores ranging from 0 to 24. Those with skin problems on examination were significantly more likely to be farmers, have had a previous skin problem, be older or live in a smaller family.

*Conclusions* This study represents the first formal documentation of skin disease prevalence in Laos and establishes the high rate of skin disease in the rural community and the associated impact these diseases have on health-related quality of life.

## Background

Skin disease is known to have a significant impact on quality of life, productivity and mental health [1, 2]. This is true of skin diseases with both an infective aetiology [3] as well as inflammatory dermatoses. In fact, the Global Burden of Disease (GBD) Study 2013, found skin disease to be the fourth (non-fatal) cause of disability worldwide [4]. It is reasonable to predict that the impact of any skin disease will be even more substantial in lower socioeconomic populations with limited access to health care and an agricultural economy.

Laos is a landlinked country in South East Asia with a population of approximately 7 million inhabitants. Over two thirds of the population rely on some form of agriculture to support their income [5]. Dermatological services are limited and exist mainly in the capital, Vientiane. Laos has a tropical monsoon climate, with the rainy season extending from May to October, followed by the cool, dry season through to February and the hot, dry season in March and April.

The purpose of this study was to assess a sample of the rural Lao population to give an indication of the prevalence of skin disease in this environment. Establishing common skin conditions in this population may assist future health planning in rural Laos and provide focus for the teaching of healthcare staff.

## Methods

Five rural villages within the Hadxaifong District of Vientiane Capital province were identified as suitable locations for the study as they had been involved in previous, non-dermatological research with the Unit. From these Hadkansa village (GPS: N 17.84041°, E 102.60451°) was selected due to the presence of a primary school and large population (1000 inhabitants).

Ethical approval was granted by the Lao National Ethics Committee for Health Research.

The study took place over four days, 15-18th May 2017, just at the very start of the rainy season, the weather had been hot and dry immediately prior to the study but heavy rains occurred during the study period.

Temporary examination rooms were created in the village temple complex and the Village Head informed the villagers in advance about the study. People were asked to attend irrespective of whether they thought they had a skin disease or not. With the assistance of the Village Head, villagers who had not attended for examination were visited in their own homes and at a local festival.

Participants gave verbal informed consent and were asked a brief series of questions about themselves and whether they had had any problems with their skin, hair or nails in the past year. Obtaining verbal consent was deemed appropriate by the Ethics committee: there were several reasons for this including literacy levels in the rural community and possible concern from participants regarding signing official documentation. For all participants under the age of 16, consent to participate was granted by their parent or legal guardian. A full body examination of their skin was then performed by one of the four dermatologists. All diagnoses were made clinically. Those who were found to have any active skin disease on examination were then asked to complete the Dermatology Life Quality Index questionnaire (DLQI) [6]. The Children’s DLQI [7] (CDLQI) was used for those under the age of 15. Both the DLQI and CDLQI were translated into Lao and the translations were piloted on several medical and non-medical native Lao speakers.

Free advice and topical treatments were given to patients where appropriate and instructions were translated into Lao. All medications remaining in the formulary after completion of the study were donated to a rural health facility. Referral to the local health services was felt to be necessary in a few cases and this was done by means of a referral letter given to the patient.

All data, including the DLQI, were recorded anonymously using the Open Data Kit programme on tablet devices. The London School of Hygiene and Tropical Medicine server was used for data storage (https://opendatakit.lshtm.ac.uk/).

The data were analysed using STATA version 15 (Stata Corporation, College Station, TX) statistical analysis software and chi square and Wilcoxon signed rank tests were performed.

## Results

Three hundred and forty participants took part in the study. Ages ranged from 4 months to 92 years with a 0.7 male/female ratio. With the exception of the age groups 15–24 years and 65 years and older, the age distribution in this sample was similar that of the population as a whole: 32.4% were aged 14 years or younger (32.7% in general population), 8% were aged 15–24 years (21.2%), 39.1% were aged 25–54 years (36.7%), 8.5%were aged 55–64 years (5.5%) and 12% were over the age of 65 years (3.9%) [5, 8].

Just under half of the participants stated farming as their primary occupation (46%, 156 participants) and 36% (122 participants) were infants, school children or students.

The size of the household ranged from 1 to 12 people, with 50% living in household of 4 or 5 people (median 5 people); the average household size in Laos is 5.3 people [5].

The summary statistics for the cohorts with and without skin disease are documented in Table 1.

**Table 1:** Summary statistics

		Total number	With skin problem	Without skin problem
Gender	Male	144 (42%)	76 (53%)	68 (47%)
	Female	196 (58%)	105 (54%)	91 (46%)
Age	< 14 yrs	109 (32%)	44 (40%)	65 (60%)
	15–64 yrs	190 (56%)	112 (59%)	78 (41%)
	> 65 yrs	41 (12%)	25 (61%)	16 (39%)
Occupation	Farmer	211 (62%)	150 (71%)	62 (29%)
	Other	129 (38%)	31 (24%)	98 (76%)
Number in household	< 3	67 (20%)	38 (57%)	29 (43%)
	4 to 6	211 (62%)	118 (56%)	93 (44%)
	> 7	62 (18%)	25 (40%)	37 (60%)
Previous skin problems	Yes	174 (51%)	124 (71%)	50 (29%)
	No	166 (49%)	57 (34%)	109 (66%)

One hundred and seventy-one participants (50%) reported having had a skin problem in the last year. Of those, 106 (62%) had used some form of treatment. When asked where they had sought advice about their previous skin problem from: 32% had seen a doctor, 26% had consulted the local pharmacy, 2% had asked the local village health worker and 2% had asked a family member.

**Fig. 1:**
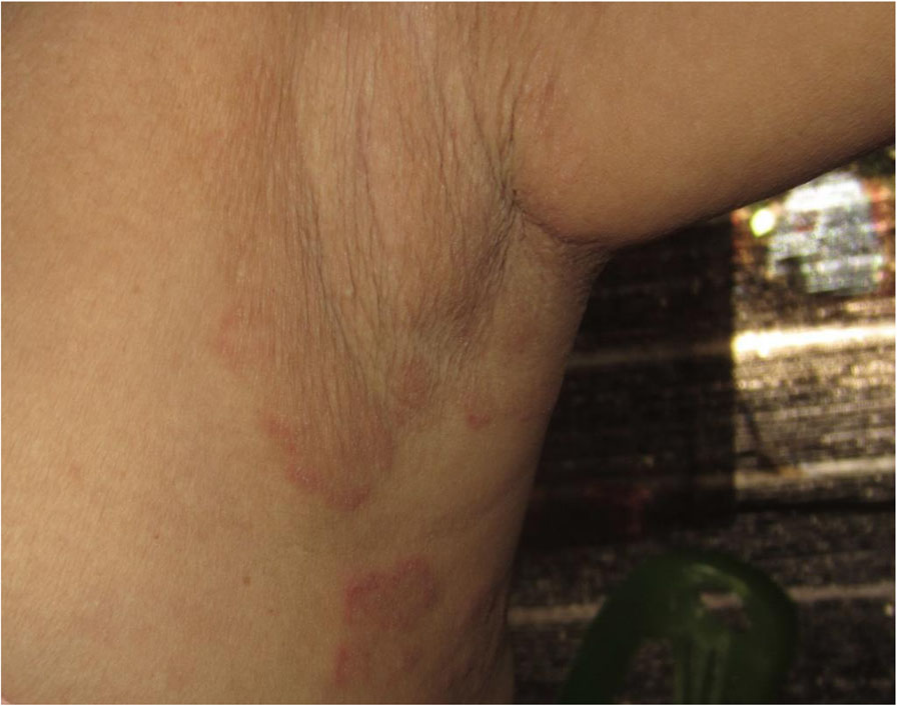
Dermatophyte infection in the axilla

On examination, just over half the participants (53%; 181 of 340 participants) were diagnosed with a skin problem. In total 258 skin conditions were recorded, incorporating a wide range of diagnoses. The six most common dermatoses were: eczema (22%; 39 people), dermatophyte infections (19%; 34 people) (Fig. 1), acne (10%; 19 people), scabies infestation (9%; 16 people), melasma (8%; 14 people) and pityriasis versicolor (4%; 8 people). Arthropod bite reactions and lichen simplex chronicus from footwear were also commonly seen (6%; 10 people and 7%; 12 people respectively). All of the skin conditions reported are listed in Table 2.

**Table 2:** Table of all skin conditions recorded

Disease	Number of cases	Percentage of participants
Infectious conditions	*61*	34%
Dermatophyte infections	34	19%
Scabies	16	9%
Pityriasis versicolor	8	4%
Viral warts	2	1%
Impetigo	1	< 1%
Pigmentary disorders	*20*	11%
Melasma	14	8%
Naevus depigmentosa	4	2%
Post inflammatory hyperpigmentation	2	1%
Inflammatory dermatoses	*99*	54%
Eczema	39	22%
Acne	19	10%
Lichen simplex chronicus	12	7%
Folliculitis	6	3%
Immersion dermatitis	5	3%
Contact dermatitis	5	3%
Pityriasis alba	4	2%
Urticaria	4	2%
Cercarial dermatitis	3	2%
Seborrhoeic dermatitis	1	< 1%
Hidradenitis suppurativa	1	< 1%
Miscellaneous skin conditions		
Arthropod bite reaction	10	6%
Traumatic wound	4	2%
Xerosis	2	1%
Keratosis pilaris	1	< 1%
Twenty nail distrophy	1	< 1%
Sacral pressure sore	1	< 1%
Accessory digits	1	< 1%
Benign tumours		
Keloid	3	2%
Epidermal cyst	1	< 1%
Dermatofibroma	1	< 1%
Malignant tumours		
Basal cell carcinoma	2	1%
Naevi and other lesions		
Vascular malformation	3	2%
Naevus sebaceous	2	1%
Infantile haemangioma	1	< 1%

**Fig. 2:**
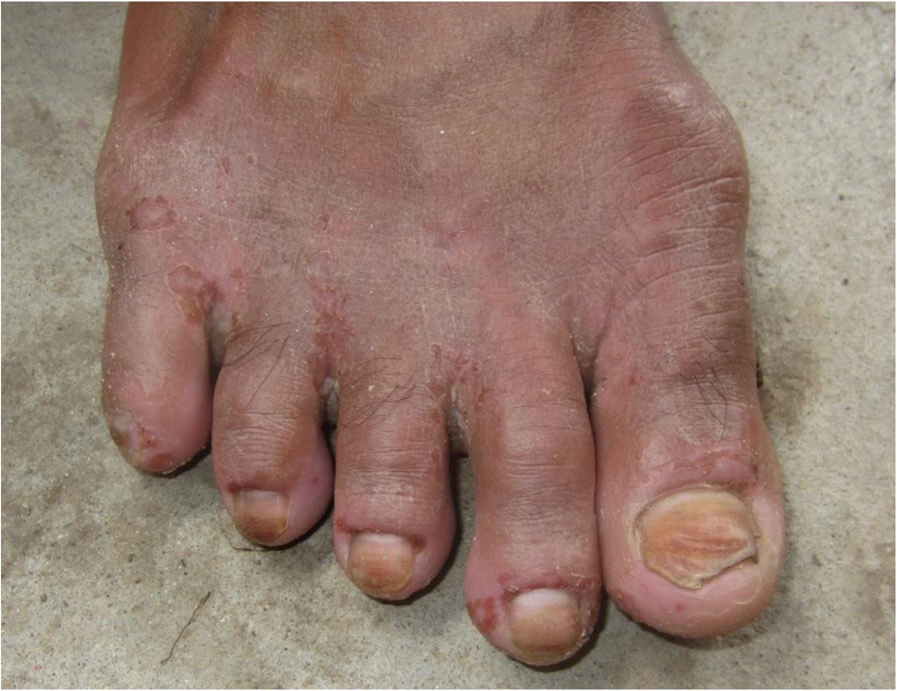
Immersion dermatitis

Of the 39 participants diagnosed with eczema; 35 had atopic dermatitis, 2 had irritant contact dermatitis, 1 discoid and 1 pompholyx eczema. Other forms of dermatitis included: 5 participants with suspected allergic contact dermatitis and 5 people with immersion dermatitis (Fig. 2) from constant exposure to water.

Of the 34 dermatophyte infections found; 16 were tinea unguium, 11 tinea pedis, 5 tinea corporis and two individual cases of tinea faciei and tinea cruris. No tinea capitis was identified.

The treatment and advice given consisted of: skin hygiene advice (29%; 52 participants); treatment from formulary (57%; 103 participants) and a visit to local health facilities recommended (2%; 4 participants).

Of the treatments given, 39% (40 participants) were given topical steroids (beclomethasone diproprionate or clobetasol proprionate depending on condition, location and severity), 30% (31 participants) were given a skin disinfectant, 11% (11 participants) were given a topical antibiotic, 16% (16 participants) were given a topical scabicide (sulphur ointment) and 23% (24 participants) were given a topical antifungal agent.

**Fig. 3:**
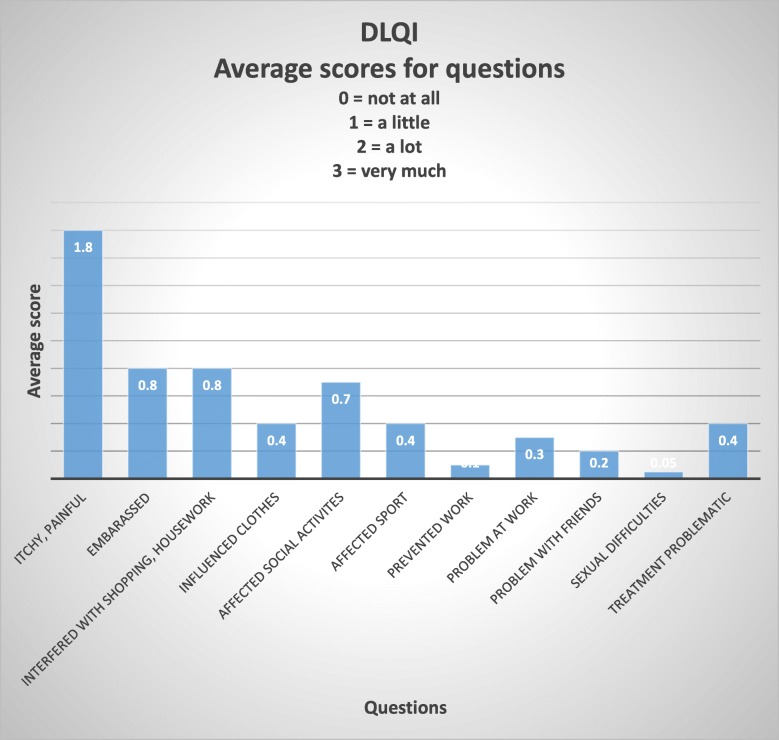
Dermatology Life Quality Index (DLQI)

**Fig. 4:**
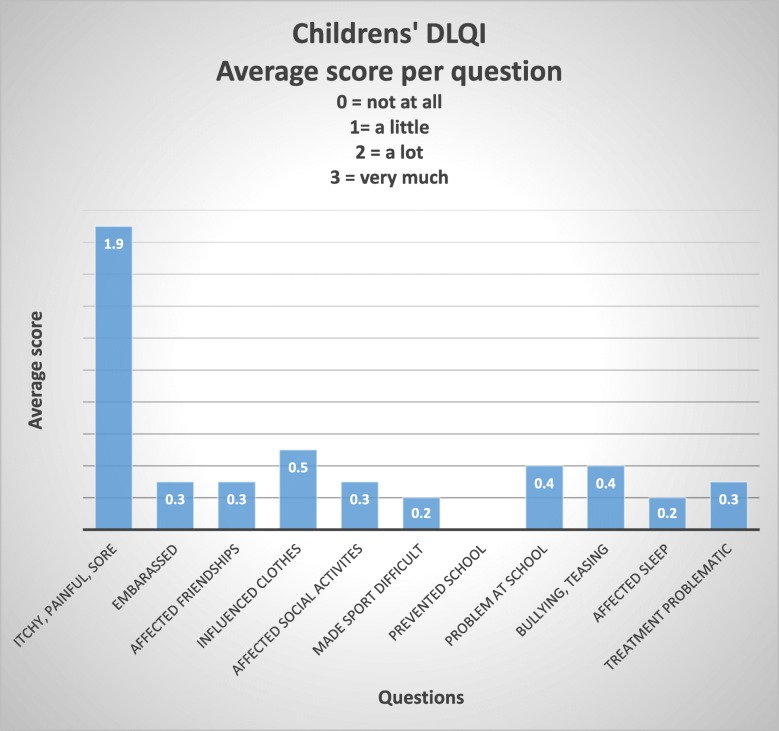
Children’s Dermatology Life Quality Index (CDLQI)

A DLQI or CDLQI was completed by 51% (91 participants) of those with skin problems. Scores ranged from 0 to 24 with a median score of 4, with 17% (20 participants) of patients completing the DLQI scoring over 10 (Table 3). A DLQI score above 10 suggests that the skin condition is having a very large impact on that individual’s life [9]. The distribution of DLQI scores is demonstrated in Fig. 3 and the CDLQI scores in Fig. 4; these figures highlight which aspects of the individual’s life are most affected by their skin condition. The diseases which resulted in DLQI scores over 10 (and therefore having the most impact in this study) were: eczema, immersion dermatitis, tinea corporis, scabies, post-inflammatory hyperpigmentation secondary to chickenpox, melasma, acne, pityriasis versicolor, cercarial dermatitis and folliculitis.

**Table 3:** DLQI scores

DLQI	Number of participants	Percentage of participants
0–1	11	12%
2–5	47	51%
6–10	18	20%
11–20	15	16%
21–30	1	1%

Chi square analysis demonstrated no significant difference between gender and the prevalence of skin disease, however it did show that farmers and/or those having had a skin problem in the previous year were significantly more likely to have a skin problem on examination (both *P* < 0.001). Wilcoxon signed rank test analysis also found that those with a skin problem on examination were significantly more likely to be older (*P* < 0.01) (mean age in the skin disease group was 36 years compared with 30 years in those without skin disease) and live in a household with fewer people (*P* < 0.04).

**Fig. 5:**
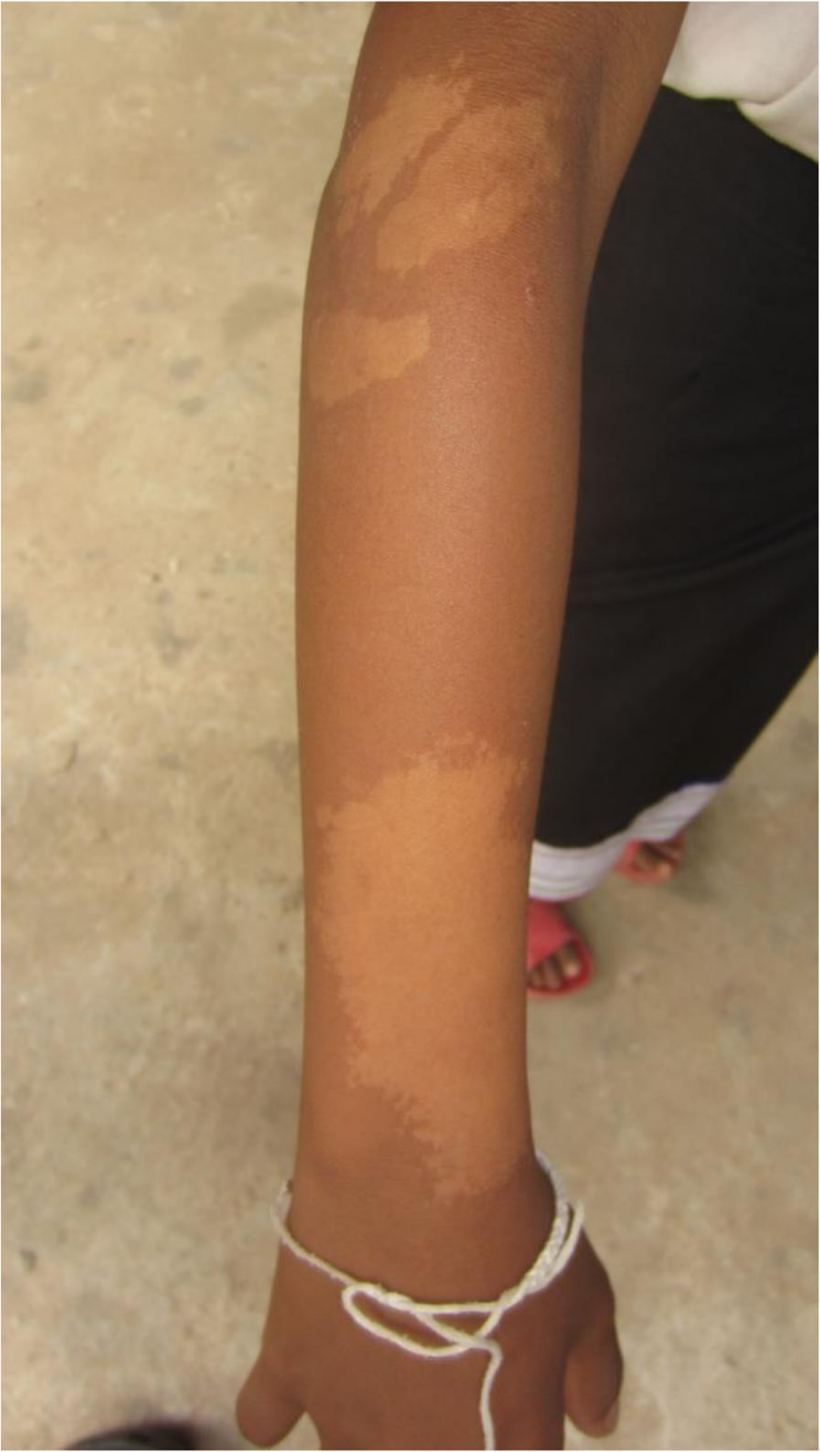
Pityriasis alba

The most common conditions seen in children (< 16 years old) were scabies, pityriasis alba (Fig. 5) and eczema. In farmers, the most common conditions were eczema, tinea unguium and tinea pedis, acne and pigmentary disorders.

## Discussion

The aim of this study was to assess skin disease prevalence in a rural Lao population. We have documented the skin diseases found in a rural Lao population and made an assessment of the resulting impact on quality of life. It is important to note however that this study used participants from only one village and as a result, while the findings shine light on the more common skin diseases in these communities, the data does not allow for an estimation of the overall prevalence of skin disease in rural Laos.

This study found a high prevalence of skin conditions (53%) with eczema, dermatophyte infections, acne, scabies infestation, melasma, pityriasis versicolor, arthropod bite reactions and lichen simplex chronicus being the most common. The study took place over a short time period and the majority of the available villagers were assessed. Having four dermatologists working in close proximity meant villagers could be assessed rapidly and any difficulties in diagnosis could be dealt with by consensus. Using the DLQI enabled a formal assessment to be made regarding the associated impact of any skin disease on an individual’s life.

Very few community, epidemiological studies of the prevalence of skin disease in South East Asia exist. Mey et al. (2015) [10] identified common dermatoses and the impact of skin disease on participants in a variety of settings in Cambodia (semi-rural and semi-urban). Cambodia and Laos are neighbouring countries and have a similar socioeconomic make up. They found an extremely high prevalence rate, with all but one participant (626 in total) having some form of skin disease. The most common conditions seen were: acne, eczema, scabies, urticaria and tinea corporis. Mey et al. also looked at the amount of money spent on treatment for dermatological conditions, finding that an average 9.5% of disposable income was spent on treating the skin condition, with acne/rosacea incurring the greatest costs. In addition, 30% of their participants reported sleep loss due to their skin disease and 13% had taken time off school or work. Many similarities exist between our data and this Cambodian study. Almost all of Mey’s participants had a skin condition and their cohort was drawn from four different locations, whereas the aim of our study was to assess the prevalence of skin disease in a single community. Unlike Mey, we did not assess the financial impact of skin disease on our cohort. Unfortunately, the adult DLQI does not include a question about sleep disturbance but the childrens’ DLQI does and 33% of the children with skin disease reported sleep loss, a similar figure to Mey’s findings. Twelve percent of adults in our study had had to take time off of work because of their skin disease and in total 34% of both adult and children completing the DLQI reported that their skin condition had affected their work or school life.

Beyond South East Asia, similar skin disease prevalence studies in rural areas have shown high frequencies of skin disease, ranging from 26 to 34% in Tanzania [11, 12], 45% in India [13], 62% in Nepal [14], 67% in Ethiopia [15] to 86% in Egypt [16]. In these studies, communicable aetiologies accounted for the majority of skin disease with dermatophyte infections, ectoparasitic skin diseases (scabies and pediculosis) causing the greatest burden of disease. Eczema, pityriasis alba, acne and pigmentary disorders accounted for the other leading causes of skin disease in these studies.

The results of this study echo the findings of previous work on skin disease prevalence in rural areas of lower to middle income countries [9–14]. Whilst high rates of dermatophyte infections were found, the majority (78%) were tinea unguium and tinea pedis, compared to tinea corporis and tinea capitis which were much more prevalent in other studies [10, 12]. No cases of pediculosis were found in our cohort despite this being common in some of the other prevalence studies [13, 14]. Eczema and scabies are a common feature of almost all skin disease prevalence studies in rural, lower socio-economic setting; the concern with both of these conditions is the propensity to lead to infection. The risk of group A streptococcal infections and subsequent acute post-streptococcal glomerulonephritis resulting from scabies is a particular concern [17]. Developing an algorithm for identifying and successfully treating both of these conditions in the rural setting could have a significant impact on the health and quality of life of these communities.

The lower rates of infectious skin diseases in both this study and Mey et al.’s study from Cambodia, compared with data from Africa and India is very interesting. There may be many reasons for this such as overcrowding, the condition of housing, occupation, etc. but one key difference is possibly access to water. Further work would be required to investigate this discrepancy.

Two studies report on the prevalence of eczema in schoolchildren in the Lao capital, Vientiane [18, 19]. Both studies used the ISAAC questionnaire to determine the prevalence of asthma, rhinitis and eczema and but no skin examination was performed. The studies asked schoolchildren aged 13–14 years and the parents of schoolchildren aged 6-7 years to complete the questionnaire. The prevalence of eczema was reported as 7.1% in Kuroiwa et al.’s study and 11.8% in Phathammavong et al.’s study which was performed 8 years later in 2006. Only 3% (3 participants) of our school aged cohort were found to have evidence of eczema on examination. This discrepancy may be due to several factors: firstly our cohort were from a rural area, rather than the urban location used in the two previous studies. Secondly, in our study the diagnosis of eczema was based on skin examination rather than a questionnaire-based diagnosis used in the two ISAAC questionnaire studies (an itchy rash coming and going for at least 6 months). Finally, our study only considered skin disease at one time point whereas the questionnaire studies asked about eczema at any time point. These differences may account for the discrepancy in prevalence of eczema found in these cohorts.

The demographics of the participants in this study match the population as a whole fairly closely, although there was a higher proportion of people over the age of 65 years (12% compared to 3.9%). This is most likely due to a significant proportion of the villagers aged between 15 and 64 years going to Thailand for up to two-three months at a time for work or education. This also accounts for the fact that despite significant efforts to examine the majority of the villagers, less than half of the registered number of village inhabitants were enrolled in the study. This significant fluctuation in population was not fully appreciated prior to the study taking place and is apparently common in most rural areas where there is easy access to Thailand. Unfortunately, this affects the generalizability of our data to the rural population as a whole. It is not clear how this issue could be fully addressed as, with the exception of academic holidays, the time periods when individuals return to the village are determined individually by the villagers themselves. It is important to note that as a result of this missing cohort of young and middle-aged individuals, our results are likely to be biased towards older and perhaps less well individuals.

This study was performed at the very start of the rainy season, the weather up unto this point had been very hot and dry. The impact of the rainy season, with increased exposure to water and mud and increased humidity, would be interesting to assess on the prevalence of skin disease in this cohort. Despite the dry weather, several cases of immersion dermatitis and cercarial dermatitis were reported, it would be reasonable to expect the number of cases of these conditions to increase with wetter conditions.

Whilst only 46% of the participants stated their occupation as farming, the vast majority are involved in agriculture to some degree, including the children. Traditionally, all able members of the family will help with the rice planting and harvesting, which involves many hours spent in wet, muddy ground. Fishing and hunting for frogs and other reptiles for food is another common activity and again involves repeated exposure to water and mud. The influence of these activities on the development of skin disease may be significant but also the impact of these essentially occupation-related dermatoses on performing these activities should be considered.

Our results found that having had a previous skin problem made participants significantly more likely to have a skin problem on examination during the study, this is perhaps unsurprising. The results also demonstrated that people registering their occupation as farmers were also significantly more likely to have a skin problem. Additionally, the cohort with skin disease were significantly older than those without. The data also revealed that participants with skin disease were significantly more likely to live with fewer people at home; this is a slightly surprising finding. However, the median number of people in the household was 5 in both groups. With the exception of scabies, there were no cases of skin diseases classically associated with overcrowding/close contact such as tinea capitis and pediculosis, which may help explain why living in a household with more people was not a risk factor for skin disease in this cohort.

The aim of this study was to assess the prevalence of skin disease in a rural Lao community. Whilst the village used was rural and agriculture was the main occupation of the majority of participants, the village is sited within the Vientiane Capital province and is only around 10 km from the capital city. As mentioned the vast majority of medical expertise, especially dermatological, exist in the capital Vientiane, and is therefore in reach of our cohort of villagers. Assessing the prevalence of skin disease in much more rural communities where there is very limited access to medical care, may yield different results. A further question regarding how representative our sample is, is the nature of the village chosen. Hadkansa village is large, with a documented population of 1000, although as discussed above the actual day-to-day population is far lower. In addition, it has a primary school and already had links with LOMWRU. These three points were the main reasons for choosing the village. As the study involved dermatologists coming to Laos from overseas, it was time-limited so we needed a large population so that as many villagers as possible could be examined. The presence of the primary school meant we could also examine primary aged children and as the study took place during weekdays, we would not have had access to this cohort otherwise. Finally, the links with LOMWRU were vital to ensure that we could organize and perform the study within the available timeframe.

**Fig. 6:**
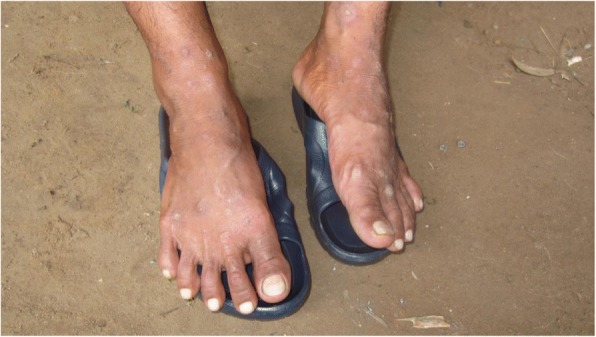
Cercarial dermatitis

The Dermatology Life Quality Index (DLQI) questionnaire was completed by 51% of the participants with a skin disease. The DLQI was chosen as an internationally recognized assessment tool, guaranteeing the translatability of the data. The decision to ask a participant to complete the DLQI was made by the clinician examining the patient, based on the skin condition found. The consensus prior to initiation of the study was to request a DLQI on any participant with an active skin condition. The relatively low percentage of completed DLQI questionnaires compared to the number of participants with a skin disease may be due to several factors: variability between the clinicians as to which conditions warranted a DLQI to be performed, participants being unwilling to complete the questionnaire, or possibly failure to input the data properly. The DLQI was recorded by two Lao clinicians on an ODK device in a separate room and it may be that some participants did not attend this station despite being requested to. Unfortunately, reasons for not completing the DLQI were not recorded. The DLQI was translated into Lao (using both the English and Thai versions as a guide to translation) and piloted on a small number of individuals. Of the DLQI scores recorded, the results demonstrated that the majority of skin conditions found had a small to moderate effect on the participant’s quality of life [8]. However, in 22% (20 people), the skin condition impacted the participant’s life to a very large or extremely large degree, according to the DLQI result rating system [8]. In this study, 3 (9%) participants were found to have cercarial dermatitis (Fig. 6) but only 2 completed the DLQI questionnaire, however their scores (12 and 17) indicate that this condition has a very large impact on their health-related quality of life. The high scores seen for both immersion and cercarial dermatitis are a concern as both of these diseases are associated with water and mud exposure from farming/fishing. Farming and fishing provide food and income to people living in rural areas: diseases resulting from these activities, that may also impair an individual’s ability to participate, may have a significant impact on both the financial and physical well-being of that individual and their family. As this study was performed at the very beginning of the wet season, it can be postulated that the incidence of cercarial and immersion dermatitis will increase during the wet season when there is increased exposure to water and mud. Introducing a programme to raise awareness and implement effective management of these conditions, could have a substantial impact on the disease burden in these areas.

There are several limitations to this study. Firstly, the translation of the DLQI/CDLQI into Lao was only piloted on a small number of individuals and its validity has not been assessed. Secondly, the study took place at only one time point in the year; given the significant climate and environment change between the wet and the dry seasons, it would be very useful to have data on skin disease during the wet season. Thirdly, just over half of the participants with a skin condition completed the DLQI: achieving a higher rate of DLQI completion would have led to more useful information. As mentioned, a significant proportion of the study village population travel to Thailand for work or education, which meant that despite best efforts, it was not possible to examine the majority of the registered village inhabitants. And finally, although the village studied is a rural community it is still very close to the capital city. Conducting this research in even more rural communities, perhaps to the north and to the south, would provide a more comprehensive documentation of the prevalence of skin disease in rural Laos.

## Conclusion

We have assessed the prevalence of skin disease in a rural Lao population and assessed the association of these diseases with the health-related quality of life of those affected. Our results echoed many of the findings from previous studies in other rural communities except that the rate of infectious skin diseases was lower in our study. The DLQI results confirmed that skin disease in this community can be associated with significant adverse health-related quality of life scores and of particular interest is the impact caused by cercarial and immersion dermatitis. Both of these diseases are associated with exposure to water, which is a hazard for those involved with farming and fishing, as many in this community are. The concern is that the number of these conditions might increase during the rainy season, causing an even greater burden of disease. Interventions to raise awareness and manage these conditions could result in a significant improvement in productivity and quality of life.

## Abbreviations

DLQI: Dermatology Life Quality Index
CDLQI: Childrens’ Dermatology Life Quality Index
